# ST8SIA6-AS1 Promotes the Epithelial-to-Mesenchymal Transition and Angiogenesis of Pituitary Adenoma

**DOI:** 10.1155/2022/7960261

**Published:** 2022-06-22

**Authors:** Zuowei Li, Chengcheng Zhang, Xin Zong, Zhao Wang, Rong Ren, Lei Wang, Ping Sun, Chunmei Zhu, Mingxia Guo, Guizhen Guo, Guo Hu, Ya'nan Wu

**Affiliations:** ^1^Shandong University of Traditional Chinese Medicine, Jinan, 250000 Shandong, China; ^2^Department of Encephalopathy, Affiliated Hospital of Shandong University of Traditional Chinese Medicine, Jinan, 250000 Shandong, China; ^3^Health Management Center, People's Hospital of Chengyang, Qingdao, 266109 Shandong, China; ^4^Department of Traditional Chinese Medicine, People' Hospital of Chengyang, Qingdao, 266109 Shandong, China; ^5^Department of Oncology, Chengyang District Peoples Hospital, Qingdao, 266000 Shandong, China

## Abstract

To investigate the effect of long noncoding RNA ST8SIA6-AS1 on the epithelial-to-mesenchymal transition (EMT) and angiogenesis of pituitary adenoma and its possible mechanism. The expression levels of ST8SIA6-AS1 and HOXA9 in noninvasive pituitary adenoma and invasive pituitary adenoma were detected using qRT-PCR. sh-ST8SIA6-AS1 transfection silenced the expression of ST8SIA6-AS1 in GH3 and GTI-1 cells. The effects of ST8SIA6-AS1 on the proliferation, invasion, angiogenesis, and EMT of GH3 and GTI-1 pituitary adenoma cells were detected. The migration ability of cells was detected through scratch assay. Dual luciferase analysis verified the targeting relationship between ST8SIA6-AS1 and miR-5195-3p. ST8SIA6-AS1 and HOXA9 were highly expressed in invasive pituitary adenoma. In pituitary adenomas, miR-5195-3p directly targeted HOXA9. miR-5195-3p is the target gene of ST8SIA6-AS1. ST8SIA6-AS1 knockdown inhibited the proliferation, invasion, angiogenesis, and EMT of pituitary adenoma. HOXA9 expression mediates the biological effect of ST8SIA6-AS1. ST8SIA6-AS1 targets miR-5195-3p to regulate the expression of HOXA9 and promote the EMT of pituitary adenomas.

## 1. Introduction

Pituitary adenoma is a common intracranial tumor with different biological behaviors because of its cell proliferation and endocrine characteristics [1]. Although the malignancy of pituitary adenoma is rare, some pituitary adenomas can infiltrate the tissues around the sellar region, destroy the normal structure, infiltrate the vascular wall, and invade the cavernous sinus and the surrounding brain tissue [2]. The occurrence of pituitary adenoma may be related to the regulation of the cell cycle, the expression of oncogenes, and the deletion of tumor suppressor genes [3, 4]. However, its pathogenesis remains unclear [5].

Epithelial-to-mesenchymal transition (EMT) is characterized by decreased endothelial adhesion factors and increased cytoskeleton contraction proteins in tumor cells [6–9]. Thus, cell morphology is transformed into mesenchymal cells, and epithelial cells are separated from the basement membrane [8]. Thus, it prepares tumor cells for migration [10, 11]. Vasculogenic mimicry (VM) is a blood supply pathway that exists in highly malignant tissues such as ovarian, breast, and liver cancers. This unusual blood supply channel is formed by plastic malignant cells deforming themselves with the extracellular matrix without the involvement of endothelial cells. Therefore, it is necessary to further study the molecular mechanism of angiogenesis mimicry. Through EMT, tumor cells have acquired high migration ability. More importantly, it has been reported that tumor cells undergoing EMT are more prone to angiogenic mimicry. Previous studies on EMT mostly focused on protein levels. With the continuous progress of gene sequencing technology, noncoding RNA has been found to play an important role in the metastasis of tumor cells. Long noncoding RNAs (lncRNAs) cover most of the noncoding information in human DNA [12]. lncRNAs comprise more than 90% of the entire genome, and they constitute a wide and complex group of molecules [13]. An increasing number of studies have proved that lncRNAs can undergo molecular exchange during cell differentiation, migration, and apoptosis and change the cell state by changing the gene expression pattern. lncRNAs can exert their functions through *cis*- or *trans*-regulation [14]. In addition, lncRNAs can interact with protein or mRNA molecules, thus affecting their stability [15, 16]. Importantly, lncRNAs play an important role in pituitary adenoma. ST8SIA6-AS1 reportedly plays an important role in other tumors. In specific, ST8SIA6-AS1 promotes hepatocellular carcinoma by absorbing miR-5195-3p to regulate HOXB6 [17]. In breast cancer, ST8SIA6-AS1 promotes proliferation, migration, and invasion through the p38 MAPK signaling pathway [18]. However, the role of ST8SIA6-AS1 in pituitary adenoma remains unclear.

Many studies have shown the importance of miRNAs in tumor biological processes, including tumor genesis, progression, and metastasis [19]. miRNAs may play a larger role in pituitary adenoma tumorigenesis than previously expected [20]. Recent studies have found that the HOXA9 gene encodes an important transcriptional regulator in embryonic development, hematopoietic regulation, and tumor development [21–23]. The regulatory mechanism of HOXA9 expression must be elucidated to understand the pathogenesis of tumors, especially pituitary adenoma.

This study is aimed at comparing the expression levels of ST8SIA6-AS1 and HOXA9 in invasive pituitary adenoma and noninvasive pituitary adenoma tissues. The effects of ST8SIA6-AS1 on the proliferation, invasion, and migration of pituitary adenoma cells were further studied, and the possible mechanism was explored.

## 2. Methods

### 2.1. Clinical Organization Information

A total of 30 specimens were selected for pituitary adenoma resection in the neurosurgery department of our hospital from 2019 to 2020. All specimens in this study were discovered for the first time, and no radiotherapy or chemotherapy was received. MR scans were all large adenomas (diameter > 1 cm) and had complete clinical data. The specimens were classified and staged in accordance with the standard of Wilson's modified Hardy classification system [24–26]. Fifteen cases were in the invasive pituitary adenoma group, among whom 10 were males and 5 were females. The age of the patients was in the range of 31–69 years, with an average age of 52.6 years. Fifteen cases were in the noninvasive pituitary adenoma group, among whom 9 were males and 6 were females. The age of the patients was in the range of 29–76 years, with an average age of 55.7 years. No difference in general clinical information was found between the two groups. Tumor aggressiveness was judged according to the following criteria: [1] Hardy–Knosp classification, grade III or above, and C-E stage are considered aggressive pituitary adenomas; [2] tumor cells in the sellar dura and adjacent bone are pathologically confirmed; and [3] tumor surrounding the bilateral internal carotid arteries is examined through imaging. Patients who meet any of the above three items were classified as aggressive and the rest as noninvasive. Fresh isolated specimens were taken in the operating room, and each specimen was maintained sterile. RNase contamination was strictly prevented during the entire process. The specimens were placed in a liquid nitrogen tank and stored for RT-PCR analysis. This study was approved by the Ethics Committee of Affiliated Hospital of Shandong University of Traditional Chinese Medicine and in line with Declaration of Helsinki. Informed consent of the patients was obtained.

### 2.2. Cell Culture

Pituitary tumor cell lines GH3 and HP75 were purchased from American Type Culture Collection (ATCC, Manassas, VA, USA) and cultured in the 1640 medium containing 10% FBS (Gibco, Life Technologies, Rockville, MD, USA). The cells were wetted in an incubator containing 5% CO_2_ at 37°C. When the cells were in the logarithmic growth phase, 3 × 10^5^ cells per dish were seeded and spread on a 60 mm culture dish. On the second day, when the cell coverage rate reached 80%, the culture was subcultured.

### 2.3. Cell Transfection

The transfection solution was mixed in accordance with Lipofectamine2000 instructions. Finally, sh-NC and sh-ST8SIA6-AS1 were transfected into the cells. Cultivation was performed in a 37°C, 5% CO_2_ incubator, with three replicate holes in each group. After 24 h, cells were collected to extract RNA. The same method was used to transfect miR-NC, miR-5195-3pmimics, and anti-miR-5195-3p.

### 2.4. qRT-PCR

Every 100 mg of pituitary tumor tissue samples was added with 1 mL of TRIzol reagent and fully lysed on ice. RNA was extracted from the transfected cells. In accordance with the SYBR Green PCR kit instructions, the synthesized cDNA was used as a template to perform a qPCR reaction on a fluorescent quantitative PCR machine. The PCR reaction system was composed of 0.2 *μ*L of template cDNA, 0.6 *μ*L of primer, and 5 *μ*L of SYBR Green PCR Mix. U6 served as an internal reference. The primer design was as follows: ST8SIA6 gene upstream primer: 5′-TCCTGATTCAGTGGCATGGT-3 ′, downstream primer: 5′-AGGGTTTCTTCGGTCGTCAT-3′; miR-5195-3p gene upstream primer: 5′-TAGCAGACTCTTATGATG-3′, downstream primer: 5′-TGGTGGAGTCGTCGTG-3′; and HOXA9 gene upstream primer: 5′-ATGCTTGTGGTTCTCCTCCA-3′, downstream primer: 5′-AGTTGGCTGCTGGGTTATTG-3′; the upstream primer of U6 is 5′-CTCGCTTCGGCAGCACA-3′, and the downstream primer is 5′-ACGCTTCACGAATTTGCGT-3′; and internal reference GAPDH upstream primer: 5′-GACAACAGCCTCAAGATCATCA-3′, downstream primer: 5′-TGAGTCCTTCCACGATACCAA-3′. DEPC water was added to a total reaction volume of 10 *μ*L. The PCR reaction conditions were as follows: 95°C for 2 min, followed by 95°C for 5 s, 60°C for 30 s, and 72°C for 30 s, for 40 cycles. The cycle threshold (Ct) value of each target gene was obtained by qRT-PCR amplification. The expression of the target gene was analyzed using the relative quantitative method of 2^-*ΔΔ*^CT.

### 2.5. Dual Luciferase Reporter Gene

The HOXA9 or ST8SIA6-AS 3′UTR end containing miR-5195-3p target sequence was obtained by PCR cloning. It was inserted into the pGL3 plasmid luciferase reporter gene downstream the restriction site. The plasmid was named “WT-HOXA9” or “ST8SIA6-AS-3′UTR.” The synthetic HOXA9 or ST8SIA6-AS 3′-UTR sequence with mutations was inserted into the NheI and XhoI restriction site downstream of the luciferase reporter gene of the pGL3 plasmid. MUT-HOXA9 or ST8SIA6-AS-3′UTR served as a negative control. WT/Mut-HOXA9 or ST8SIA6-AS3′UTR with miR-5195-mimic/miR-NC was transfected into cells. Then, 100 *μ*L of PBL was added after 48 h of transfection. After shaking at room temperature for 15 min, 20 *μ*L of the product was added to an equal volume of LAR II solution to detect fluorescence intensity. After finishing, 20 *μ*L stop solutions were added. The fluorescence intensity was tested again, and the relative fluorescence intensity was calculated.

### 2.6. EdU Experiment

The EdU solution was diluted in the ratio of 1 : 1000 cell medium. When the fusion degree of cells reached 50%–80%, the fluid was discarded. The culture was added with 100 *μ*L of 50 *μ*mol/L EdU medium to culture for 2 h and then 100 *μ*L of 4% paraformaldehyde PBS cell fixation solution. The cells were cultured at room temperature for 15–30 min, added with 2 mg/mL glycine, and then cultured for 10 min. The cells were rinsed with PBS twice, and then, the supernatant was discarded. The cells were added with 100 *μ*L of 0.5% TritonX-100 PBS penetrant, rinsed with PBS once, added with 100 *μ*L of 1 × Apollo dyeing reaction solution, and then cultured at room temperature for 30 min without light. The cells were rinsed with PBS once and added with 100 *μ*L of DAPI reaction solution. The cells were cultured at room temperature for 30 min without light. Then, 100 *μ*L of the penetrant was added to rinse three times. The DAPI reaction solution was eluted and photographed under a fluorescence microscope.

### 2.7. Proliferation Experiment

A single-cell suspension was prepared from the culture medium. A single-cell suspension with the concentration of 3 × 10^3^/100 *μ*L was prepared by counting and adjusting the cell concentration. Each well of the 96-well plate was added with 100 *μ*L of cell suspension. Each group had six duplicate holes. The sticking time was set to 0 h. After 48 h, the 96-well plate was removed, and 10 *μ*L of CCK-8 reagents was added to each well. The cells were incubated in the cell culture chamber for another 2 h in the dark. The absorbance value (*A*) at the wavelength of 450 nm was measured with a microplate analyzer.

### 2.8. Transwell

At 24 h after transfection, a single-cell suspension was prepared with serum-free culture medium. The cell concentration was adjusted to 5 × 10^5^ cells/mL. The upper and lower chambers of each Transwell compartment were added with 200 *μ*L of cell suspension and 200 *μ*L of cell suspension, respectively. The chamber was placed in a 24-well plate. In the invasion experiment, Matrigel glue was spread in the Transwell chamber in advance. The 24-well plates were placed in the incubator and then cultured at 37°C and 5% CO_2_ for 48 h. The compartment was removed and then washed with PBS for three times. The cells in the upper interior layer were carefully wiped with the cotton swab, immobilized with 95% ethanol for 10 min, and then stained with 4 g/L crystal violet. The cells moved to the outer layer of the microporous membrane were observed under an inverted microscope. The number of cells in 10 fields was randomly counted under 200x field. The average number of cells in each field was calculated, and the experiment was repeated for three times.

### 2.9. Scratch Test

The transfected cells were inoculated in 6-well plates and cultured for 24 h. The medium was replaced with serum-free medium. When the cell density reached 80%–90%, vertical lines were drawn forcefully with the head of a spear compared with a ruler. After rinsing the cells with PBS for three times, the cells were cultured in serum-free medium and photographed according to the designed time points. Each group was repeated with three wells, and the experiment was repeated three times.

### 2.10. Xenograft Tumor Model

The nude mice were purchased from Beijing Vital River Laboratory Animal Technology Co., Ltd. and stored in SPF sterile purification room for 5–7 weeks. sh-NC and sh-ST8SIA6-AS pituitary adenoma cells with exponential growth were mixed with an appropriate amount of PBS solution to form 1 × 10^7^/mL cell suspension. At the age of 6 weeks and the body weight of nude mice was about 20 g, 0.2 mL of cell suspension was injected subcutaneously into the right side of each nude mouse. 10 d into tumor: tumor volume was measured once a day (*V*). *V* = *AB*^2^/2 (*A* is the largest diameter of the tumor, and *B* is the smallest diameter of the tumor). After 4 weeks, mice were sacrificed by carbon dioxide-releasing devices, and the tumor was collected. The euthanasia box was not filled with CO_2_ in advance before the nude mice were placed in. When the equipment was ready, the nude mice were placed. The chamber was filled with CO_2_ at a rate of 10% of the volume of the euthanasia chamber per minute. The animal was assured to be steady and breathing with pupils dilated. Close the CO_2_: the animal was observed for another 2 min and confirmed whether or not dead. The tumor volume was calculated and weighed. This study was approved by the Ethics Committee of Affiliated Hospital of Shandong University of Traditional Chinese Medicine.

### 2.11. Immunohistochemical Analysis

HOXA9 antibody was purchased from Abcam. SP kit, diaminobenzidine chromogenic agent, and antigen repair solution were provided by Beijing Zhongshan Technology Co., Ltd. Fresh specimens were fixed with formaldehyde. Conventional paraffin embedding: continuous sections with a thickness of 4 *μ*m were sectioned. The experimental procedure was carried out following the instructions of the SP kit. The concentration of primary antibody was 1 : 100. In accordance with the Remmele–Seore standard, the percentage of positive cells in the total number of tumor cells was divided into three grades: tumor cell nucleus staining < 10% was (-), 10%–50% was (+), and >50% was (++).

### 2.12. Statistical Analysis

SPSS 22.0 software was used for statistical analysis. Measurement data were expressed as mean ± standard deviation. The *t* test was used for comparison between the two groups and for comparison within the two groups. Comparison between groups was performed by ANOVA followed by Tukey's multiple comparison test. The correlation was analyzed by the Pearson correlation coefficient. Statistical significance was considered at *P* < 0.05.

## 3. Results

### 3.1. HOXA9 Is Highly Expressed in Invasive Pituitary Adenoma

**Figure 1:**
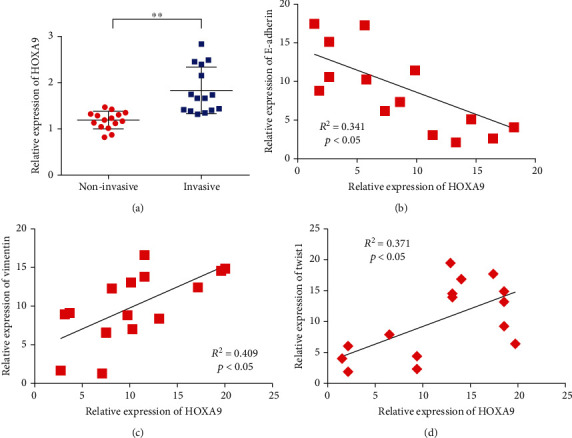
HOXA9 is highly expressed in invasive pituitary adenoma. (a) HOXA9 expression in invasive pituitary adenoma tissues was higher than that in non-invasive pituitary adenoma tissues. (b) HOXA9 expression was negatively correlated with E-cadherin coexpression. (c) The expression level of HOXA9 is positively correlated with the coexpression of vimentin. (d). HOXA9 expression level is positively correlated with Twist1 coexpression. (e) Detection of HOXA9 transfection efficiency. (f) Effects of overexpression and knockdown of HOXA9 on the proliferation ability of GH3 and GTI-1 cells. (g) Effects of overexpression and knockdown of HOXA9 on E-cadherin in GH3 and GTI-1 cells. (h) Effects of overexpression and knockdown of HOXA9 on the expression of vimentin in GH3 and GTI-1 cells. (i) Effects of overexpression and knockdown of HOXA9 on the invasion ability of GH3 and GTI-1 cells. ^∗∗^*P* < 0.01.

The expression of HOXA9 in invasive pituitary adenoma and noninvasive pituitary adenoma was detected to measure the expression of HOXA9 in pituitary adenoma. Results showed that HOXA9 expression was higher in invasive pituitary adenoma tissues than in noninvasive tissues (Figure 1(a)). In addition, further detection showed that HOXA9 expression was negatively correlated with E-cadherin coexpression (Figure 1(b)). The expression of HOXA9 was positively correlated with the expression of vimentin (Figure 1(c)). Correlation test results of coexpression of HOXA9 and tumor-related transcription factors showed that HOXA9 was copositively correlated with Twist1 (Figure 1(d)).

### 3.2. In Pituitary Adenoma, miR-5195-3p Directly Targets HOXA9

**Figure 2:**
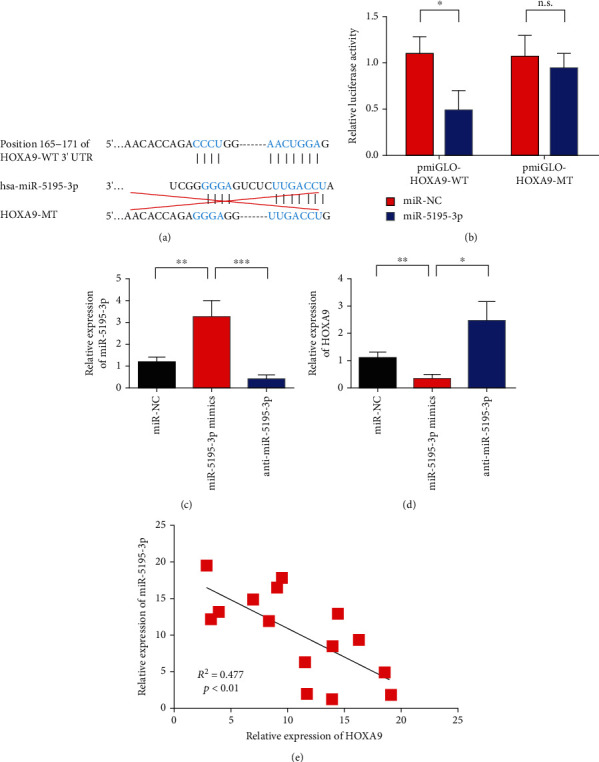
In pituitary adenoma, HOXA9 was directly targeted by miR-5195-3p. (a) Schematic diagram of the binding sites between miR-5195-3p and HOXA9. (b) The binding of miR-5195-3p to HOXA9 was verified by the dual luciferase reporter assay. (c) Verification of transfection efficiency of miR-5195-3p mimics. (d) Overexpression of miR-5195-3p inhibited the expression of HOXA9. (e) The co-expression of miR-5195-3p was negatively correlated with HOXA9. (f) Expression of miR-5195-3p in invasive pituitary adenoma tissues and noninvasive pituitary adenoma tissues was detected by qRT-PCR assay. ^∗∗^*P* < 0.01; n.s. means no statistical difference. ^∗^*P* < 0.05, ^∗∗^*P* < 0.01, and ^∗∗∗^*P* < 0.001.

miRNAs capable of regulating HOXA9 were predicted through the TargetScan website to study the regulatory mechanism of HOXA9.  Figure 2(a) shows the schematic of the binding sites between miR-5195-3p and HOXA9. Dual luciferase reporter gene assay was used to verify the binding relationship between miR-5195-3p and HOXA9 to verify whether or not miR-5195-3p specifically binds to the 3′UTR of the HOXA9 gene. The luciferase activity of the mutant plasmid was compared with that of the wild-type plasmid. Results showed that miR-5195-3p could downregulate the luciferase activity of the wild-type plasmid without affecting the luciferase activity of the mutant plasmid. Luciferase reporting assay confirmed that miR-5195-3p could specifically act on the 3′UTR of the HOXA9 gene (Figure 2(b)). After transfection with miR-5195-3p, the mRNA level of the HOXA9 gene in each treatment group was detected by real-time quantitative PCR. The mRNA level of the HOXA9 gene in the transfected miR-5195-3p group significantly decreased compared with that of the negative control group. In addition, the expression of HOXA9 was upregulated after transfection of anti-miR-5195-3p (Figures 2(c) and 2(d)). Coexpression correlation analysis results showed that the coexpression of miR-5195-3p was negatively correlated with HOXA9 (Figure 2(e)).

### 3.3. miR-5195-3p Is the Target Gene of ST8SIA6-AS1

**Figure 3:**
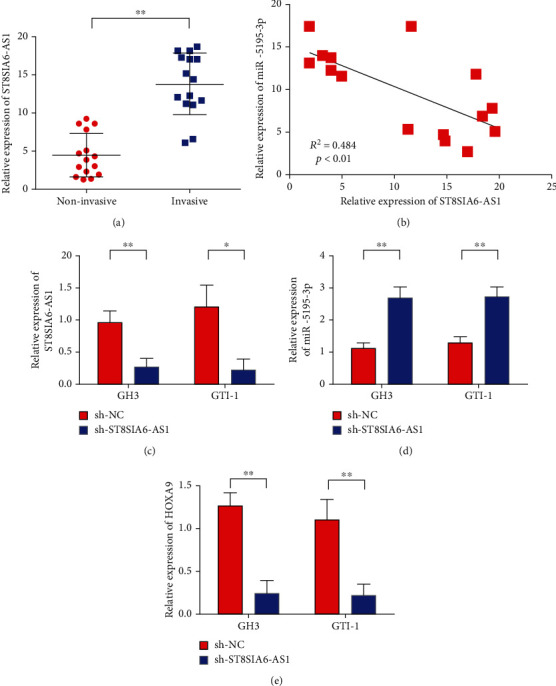
miR-5195-3p was the target gene of ST8SIA6-AS1. (a) The expression of ST8SIA6-AS1 is upregulated in invasive pituitary tumor tissues. (b) The coexpression of miR-5195-3p was negatively correlated with ST8SIA6-AS1. (c) Schematic diagram of the binding sites between miR-5195-3p and ST8SIA6-AS1. (d) The binding of miR-5195-3p to ST8SIA6-AS1 was verified by the dual luciferase reporter assay. (e) Knockdown ST8SIA6-AS1 efficient verification. (f) Knocking down ST8SIA6-AS1 upregulated the expression of miR-5195-3p. (g) Knockdown ST8SIA6-AS1 to inhibit HOXA9 expression. ^∗^*P* < 0.05, ^∗∗^*P* < 0.01.

miR-5195-3p can be regulated by ST8SIA6-AS1 [17]. We further examined the expression of ST8SIA6-AS1 in invasive pituitary tumor tissues. The experimental results of qRT-PCR showed that the expression of ST8SIA6-AS1 was upregulated in invasive pituitary adenoma tissues compared with noninvasive pituitary adenoma tissues (Figure 3(a)). Further analysis showed that the co-expression of miR-5195-3p and ST8SIA6-AS1 was negatively correlated (Figure 3(b)).  Figure 3(c) shows the schematic of the binding sites between miR-5195-3p and ST8SIA6-AS1. The binding relationship between miR-5195-3p and ST8SIA6-AS1 was verified using dual luciferase reporter gene assay to clarify whether or not miR-5195-3p specifically binds to the ST8SIA6-AS1 gene. The luciferase activity of the mutant plasmid was compared with that of the wild-type plasmid. The sh-ST8SIA6-AS1 knockdown plasmid was transfected into GH3 and GTI-1 cells for 48 h, and the level of miR-5195-3p in each treatment group was detected by real-time quantitative PCR. Experimental results showed that the expression of miR-5195-3p in the knockdown ST8SIA6-AS1 group was significantly upregulated compared with the negative control group (Figures 3(d)). These results suggest that miR-5195-3p is the downstream target gene of ST8SIA6-AS1. Further test results showed that knockdown of ST8SIA6-AS1 in GH3 and GTI-1 cells inhibited HOXA9 expression (Figure 3(e)).

### 3.4. ST8SIA6-AS1 Knockdown Can Inhibit the Proliferation, Invasion, and EMT of Pituitary Adenoma

**Figure 4:**
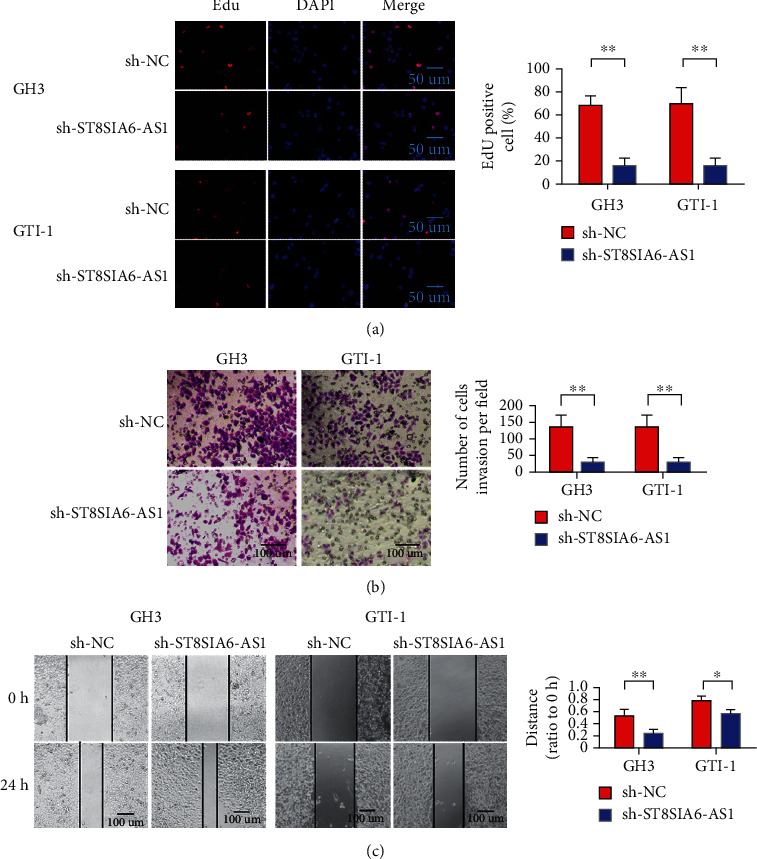
ST8SIA6-AS1 knockdown inhibited the proliferation and invasion of pituitary adenoma. (a) Knocking down ST8SIA6-AS1 can inhibit the proliferation of GH3 and GTI-1 in pituitary adenoma cells (magnification 400x). (b) ST8SIA6-AS1 knockdown inhibited the invasion of GH3 and GTI-1 pituitary adenoma cells (magnification 200x). (c) ST8SIA6-AS1 knockdown can inhibit the migration of GH3 and GTI-1 in pituitary adenoma cells (magnification 200x). (d) ST8SIA6-AS1 knockdown can inhibit the VE-cadherin in pituitary adenoma cells. ^∗^*P* < 0.05, ^∗∗^*P* < 0.01.

The changes in the proliferation, invasion, and migration of GH3 and GTI-1 cells after the downregulation of ST8SIA6-AS1 gene interference were analyzed to understand further the biological role of ST8SIA6-AS1. EDU experiment showed that, compared with the empty vector control group (sh-NC), the proliferation ability of GH3 and GTI-1 cells was significantly reduced after knockdown of ST8SIA6-AS1 (Figure 4(a)). This result indicates that knockdown of the ST8SIA6-AS1 gene can inhibit the proliferation ability of GH3 and GTI-1 cells. Transwell cell invasion assay showed that compared with the empty vector control group (sh-NC), the number of cells invaded into the lower compartment significantly reduced after ST8SIA6-AS1 knockdown (Figure 4(b)). This result suggests that ST8SIA6-AS1 knockdown can inhibit the invasion ability of GH3 and GTI-1 cells. Scratch test showed that ST8SIA6-AS1 knockdown could significantly reduce the migration ability of GH3 and GTI-1 cells (Figure 4(c)).

### 3.5. ST8SIA6-AS1 Knockdown Can Inhibit the EMT of Pituitary Adenoma Cells

**Figure 5:**
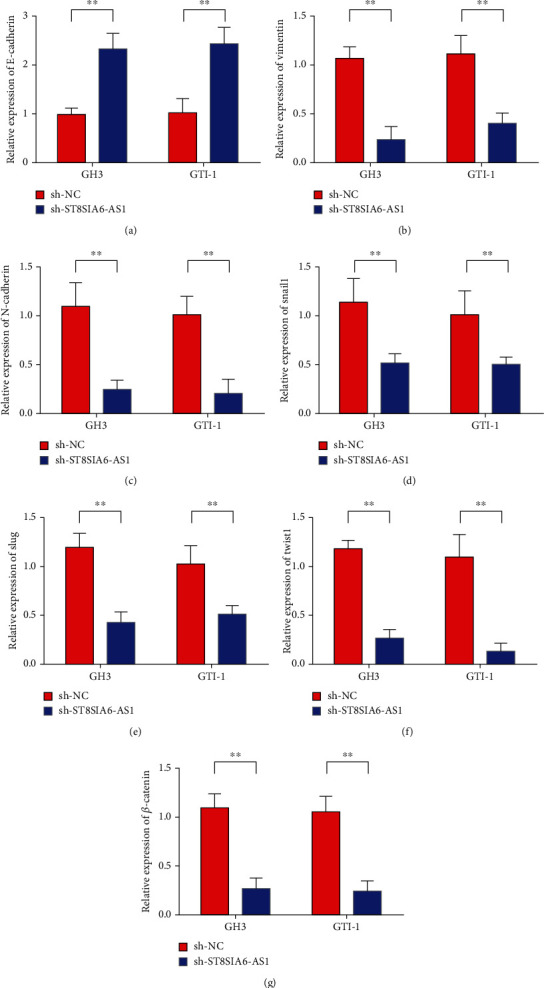
ST8SIA6-AS1 knockdown inhibited pituitary adenoma cell EMT. (a). Detection of E-cadherin expression in GH3 and GTI-1 cells. (b) Detection of vimentin expression in GH3 and GTI-1 cells. (c) Detection of N-cadherin expression in GH3 and GTI-1 cells. (d) Detection of Snail1 expression in GH3 and GTI-1 cells. (e) Detection of Slug expression in GH3 and GTI-1 cells. (f) Detection of Twist1 expression in GH3 and GTI-1 cells. (g) Detection of *β*-catenin expression in GH3 and GTI-1 cells. (g) ST8SIA6-AS1 knockdown can inhibit the expression of VE-cadherin in pituitary adenoma cells; ^∗∗^*P* < 0.01.

Changes of EMT markers in GH3 and GTI-1 cells after the downregulation of ST8SIA6-AS1 gene interference were analyzed. Detection results of E-cadherin expression in GH3 and GTI-1 cells showed that the expression of E-cadherin increased after ST8SIA6-AS1 knockdown (Figure 5(a)). The expression of vimentin in GH3 and GTI-1 cells decreased after ST8SIA6-AS1 knockdown (Figure 5(b)). In addition, ST8SIA6-AS1 knockdown inhibited the expression of N-cadherin in GH3 and GTI-1 cells (Figure 5(c)). Subsequently, changes in the expression of tumor-associated transcription factors were examined. Results showed that ST8SIA6-AS1 knockdown inhibited the expression of SNAIL1, SLUG, and TWSIT1 in GH3 and GTI-1 cells (Figures 5(d)–5(f)). Furthermore, ST8SIA6-AS1 knockdown inhibited the expression of *β*-catenin and VE-cadherin in GH3 and GTI-1 cells (Figures 5(g)). The results showed that ST8SIA6-AS1 knockdown can inhibit the EMT and angiogenesis of pituitary adenoma cells, and the changes in the above markers were consistent with the trend of superficial changes of pituitary adenoma cells.

### 3.6. HOXA9 Expression Mediates the Biological Effect of ST8SIA6-AS1

**Figure 6:**
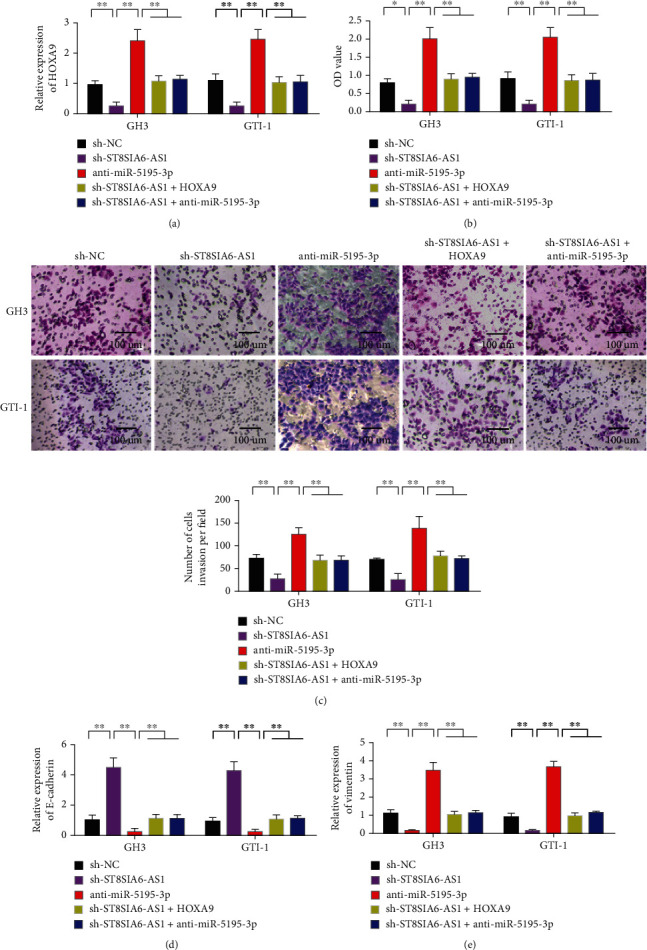
Biological effect of HOXA9 expression mediating ST8SIA6-AS1. (a) HOXA9 expression in GH3 and GTI-1 cells was detected after different treatments. (b) GH3 and GTI-1 cell proliferation was detected after different treatments. (c) Invasive detection of GH3 and GTI-1 cells after different treatments. (d) Detection of E-cadherin expression in GH3 and GTI-1 cells after different treatments. (e) Vimentin expression levels of GTI-1 and GTI-1 cells were detected after different treatments. ^∗^*P* < 0.05, ^∗∗^*P* < 0.01. Magnification 200x.

The changes in pituitary adenomas after different treatments were further analyzed to verify the ceRNA mechanism of ST8SIA6-AS1/miR-5195-3p/HOXA9. The experiment was divided into five groups: sh-NC, sh-ST8SIA6-AS1, anti-miR-5195-3p, sh-ST8SIA6-AS1+HOXA9, and sh-ST8SIA6-AS1+anti-miR-5195-3p. The expression of HOXA9 in GH3 and GTI-1 cells after different treatments was detected. Experimental results showed that the expression of HOXA9 decreased after knocking down ST8SIA6-AS1. However, the expression of HOXA9 was upregulated after the transfection of anti-miR-5195-3p. Compared with the knockdown ST8SIA6-AS1 group, the expression of HOXA9 in the sh-ST8SIA6-AS1+HOXA9 and sh-ST8SIA6-AS1+anti-miR-5195-3p groups was upregulated simultaneously (Figure 6(a)). The proliferation and invasion of ST8SIA6-AS1 cells decreased after the knockdown. However, the proliferation and invasion of the cells were upregulated after transfection of anti-miR-5195-3p. The proliferation and invasion of the sh-ST8SIA6-AS1+HOXA9 and sh-ST8SIA6-AS1+anti-miR-5195-3p groups increased compared with those of the knockdown ST8SIA6-AS1 group (Figures 6(b) and 6(c)). After different treatments, the expression of E-cadherin in GH3 and GTI-1 cells was detected, and ST8SIA6-AS1 knockdown promoted the expression of E-cadherin. However, miR-5195-3p knockdown inhibited the expression of E-cadherin. The expression of E-cadherin in the sh-ST8SIA6-AS1+HOXA9 and sh-ST8SIA6-AS1+anti-miR-5195-3p groups increased simultaneously compared with that in the knockdown ST8SIA6-AS1 group (Figure 6(d)). After different treatments, the vimentin expression levels in GTI-1 and GTI-1 cells were detected, and ST8SIA6-AS1 knockdown inhibited vimentin expression. The knockdown of miR-5195-3p promoted the expression of vimentin. The expression of vimentin in the sh-ST8SIA6-AS1+HOXA9 and sh-ST8SIA6-AS1+anti-miR-5195-3p groups decreased compared with that in the knockdown ST8SIA6-AS1 group (Figure 6(e)).

### 3.7. Downregulation of ST8SIA6-AS1 Expression Can Inhibit Tumor Growth In Vivo

**Figure 7:**
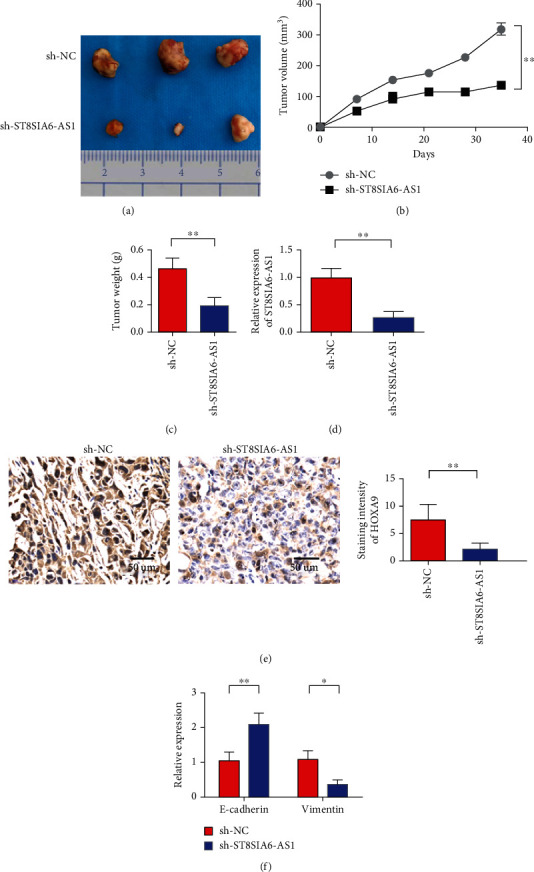
Downregulation of ST8SIA6-AS1 expression can inhibit tumor growth in vivo. (a) Downregulation of ST8SIA6-AS1 expression significantly inhibited tumor growth in GTI-1 cells in the nude mouse model. (b) Tumor volume detection. (c) Tumor weight detection. (d) Real-time PCR analysis showed that the expression of ST8SIA6-AS1 was significantly decreased in tumor tissues of nude mice after knockout of ST8SIA6-AS1. (e) Real-time PCR analysis showed that the expression of miR-5195-3p was significantly increased in tumor tissues of nude mice after knockout of ST8SIA6-AS1. (f) Immunohistochemical analysis showed that the expression of HOXA9 was significantly reduced in tumor tissues of nude mice after the knockout of ST8SIA6-AS1. (g) Real-time PCR analysis showed changes in the expression of E-cadherin and vimentin in tumor tissues of nude mice after knockout of ST8SIA6-AS1. ^∗^*P* < 0.05, ^∗∗^*P* < 0.01. Magnification 400x.

Animal experiment results showed that downregulation of ST8SIA6-AS1 expression significantly inhibited the tumor growth of GTI-1 cells in the nude mouse model (Figure 7(a)). Tumor volume detection results also showed that tumor growth rate decreased after ST8SIA6-AS1 knockdown compared with the control group (Figure 7(b)). Tumor weight also decreased after the decrease in ST8SIA6-AS1 (Figure 7(c)). Real-time PCR analysis showed that the expression of ST8SIA6-AS1 significantly decreased in the tumor tissues of nude mice after its knockout (Figure 7(d)). Immunohistochemical analysis showed that the expression of HOXA9 in the tumor tissues of nude mice significantly decreased after ST8SIA6-AS1 knockdown (Figure 7(e)). Real-time PCR analysis showed that at the tumor tissue level, the expression of E-cadherin was upregulated after the knockout of ST8SIA6-AS1, whereas the expression of vimentin decreased in nude mouse tumor tissues (Figure 7(f)). The results of animal experiments showed that the tumor growth rate decreased after ST8SIA6-AS1 knockdown. Further test results showed that ST8SIA6-AS1 knockdown upregulated the expression of miR-5195-3p in tumor tissues while downregulated the expression of HOXA9. Thus, the tumor-suppressive effect of ST8SIA6-AS1 is mediated by the ST8SIA6-AS1/miR-5195-3p/HOXA9 axis.

## 4. Discussion

The formation of pituitary tumors is a multilink process, which includes the deletion of tumor suppressor genes, overexpression of protooncogenes, and dysregulation of cell cycle and cell proliferation [27]. In tumor invasion, tumor cells separate from each other, bind with matrix components, and degrade extracellular matrix. Reduced adhesion between tumor cells is an important mechanism of tumor invasion [28–31].

Recent studies have found that the HOX gene family is closely related to the occurrence and development of various tumors. The HOXA9 gene belongs to the A group of HOX genes located on the short arm of human chromosome 7 (7p15.2) [32, 33]. It encodes a protein with a relative molecular weight of approximately 30 kD that functions as a transcription factor. The HOXA9 locus is rich in CpG islands, and changes in methylation state are associated with a variety of tumors [34]. In addition to acute myeloid leukemia, HOXA9 is also closely associated with ovarian cancer and glioma [35, 36]. In the present study, HOXA9 expression was correlated with tumor EMT characteristics. HOXA9 is highly expressed in invasive pituitary adenomas. Meanwhile, HOXA9 was negatively correlated with E-cadherin coexpression. HOXA9 was positively correlated with vimentin and Twist1. Pojo et al. [37] confirmed through cytological experiments and in vivo tumorigenetic experiments that HOXA9 can promote the survival and invasion of glioma cells and inhibit cell apoptosis. It plays a role in promoting cancer during the initiation and progression of tumors. Pojo et al. also demonstrated in vivo and in vitro that HOXA9 can upregulate Bcl-2 and lead to the temozolomide treatment resistance of patients with glioma, providing a new idea for the treatment of glioma [37].

In the present study, we used HOXA9 as the target gene. MicroRNAs that play regulatory roles were screened by TargetScan prediction and luciferase reporting assay. Results showed that miR-5195-3p can downregulate the luciferase activity of the 3′UTR vector of the HOXA9 gene, which is a microRNA that might play a regulatory role. A mutant luciferase reporter gene vector was constructed by mutating the miR-5195-3p binding site in the 3′UTR region of the HOXA9 gene predicted by TargetScan software to verify the active sites of miR-5195-3p regulating the HOXA9 gene. The cells were cotransfected with the miR-5195-3p expression plasmid. Compared with the unmutated 3′UTR vector, luciferase activity was significantly restored. These results suggest that miR-5195-3p plays a role by binding to the binding site in the 3′UTR region of the HOXA9 gene.

lncRNAs may affect the occurrence and development of pituitary adenoma. Zhang et al. first found that lncRNA MEG3 is a potential tumor suppressor for pituitary tumors [38]. Since then, researchers have studied it in other tumors, and the role and mechanism of human maternally expression gene3 (MEG3) in tumors have been clarified. Chunharojrith et al. also found that MEG3 could significantly inhibit the growth of xenograft tumor in nude mice and block the G1 phase of the cell cycle. In addition, p53 gene inactivation completely impedes the tumor inhibition of MEG3, suggesting that the tumor inhibition of MEG3 is mediated by p53 [39].

Further studies showed that miR-5195-3p is the target gene of ST8SIA6-AS1. In breast, lung, and pancreatic cancers, knockdown of ST8SIA6-AS1 leads to mitotic disorders and massive cell apoptosis [40]. ST8SIA6-AS1 plays an important role in the occurrence and development of cancer. However, the role of ST8SIA6-AS1 in pituitary adenoma has not been reported. In this study, the expression of ST8SIA6-AS1 in the GH3 and GTI-1 cell lines of human pituitary adenoma was silenced. Results showed that the silencing of ST8SIA6-AS1 significantly reduced the proliferation, invasion, and migration of GH3 and GTI-1 cells. The expression of miR-5195-3p in GH3 and GTI-1 cells was detected by real-time quantitative PCR after ST8SIA6-AS1. Results showed that silencing ST8SIA6-AS1 can upregulate the expression level of miR-5195-3p in GH3 and GTI-1 cells. Luciferase reporter gene results confirmed that miR-5195-3p is the target gene of ST8SIA6-AS1.

In this study, we investigated the relationship between ST8SIA6-AS1 and EMT and tumor angiogenic mimicry at the cellular and animal levels. Further attempts were made to reveal its potential molecular mechanism and potential as a therapeutic target. More importantly, we verified that downregulation of ST8SIA6-AS1 expression can reverse EMT and inhibit angiogenic mimicry from the perspectives of gene level, protein level, and cell biological characteristics.

## 5. Conclusion

ST8SIA6-AS1 was highly expressed in invasive pituitary adenomas. Silencing of ST8SIA6-AS1 can inhibit the proliferation, invasion, and migration of GH3 and GTI-1 cells by inhibiting EMT. The results of the mechanism study suggest that the oncogenic effect of ST8SIA6-AS1 can be realized through the miR-5195-3p/HOXA9 axis.

## Data Availability

The datasets used and/or analyzed during the current study are available from the corresponding author on reasonable request.
